# Beyond Rigler's Triad: Gallstone Ileus in the Absence of a Bilioenteric Fistula

**DOI:** 10.7759/cureus.108147

**Published:** 2026-05-02

**Authors:** Luis F Ochoa, Vanessa Galvan, Ileana P McCall, Katia Ramirez, Karen D Zambrano, Rishita Dave, Hans F Adolphs, Juan M Jimenez, Walter V Jovel Suazo, Sukhanya Subramaniam, Veronica Contasti, Katherine S Trejos Guzman

**Affiliations:** 1 Surgery, Hospital General ISSSTE (Instituto de Seguridad y Servicios Sociales de los Trabajadores del Estado) Presidente General Lázaro Cárdenas, Chihuahua, MEX; 2 Surgery, IMSS (Instituto Mexicano del Seguro Social) Regional General Hospital No. 1, Ciudad Obregon, MEX; 3 Surgery, Universidad Evangélica de El Salvador, San Salvador, SLV; 4 General Medicine, Universidad Xochicalco, Tijuana, MEX; 5 Internal Medicine, University of Medicine and Health Sciences, Basseterre, KNA; 6 General Medicine, Universidad del Norte, Barranquilla, COL; 7 Surgery, Universidad Autónoma de Chihuahua, Chihuahua, MEX; 8 Surgery, Universidad de El Salvador, San Salvador, SLV; 9 Surgery, Chettinad Hospital and Research Institute, Kelambakkam, IND; 10 Surgery, Albert Einstein College of Medicine, New York City, USA; 11 Surgery, Universidad Nacional Autónoma de Nicaragua, Managua, NIC

**Keywords:** cholecysto-enteric fistula absent, cholelithiasis, gallstone diseases, gallstone ileus, small bowel enterotomy

## Abstract

Gallstone ileus is an uncommon cause of mechanical intestinal obstruction, classically associated with bilioenteric fistula formation that enables gallstone migration into the gastrointestinal tract. Its incidence increases among elderly patients and is associated with significant morbidity due to delayed diagnosis. Obstruction typically occurs at the terminal ileum, and diagnosis traditionally relies on Rigler’s triad: intestinal obstruction, pneumobilia, and an ectopic gallstone.

Gallstone ileus without a bilioenteric fistula is an exceptionally rare presentation that challenges conventional diagnostic paradigms. The absence of pneumobilia or identifiable fistulous communication may obscure clinical suspicion and delay treatment. Proposed mechanisms include transpapillary migration through the ampulla of Vater, transient biliary dilation, spontaneously closed microfistulas, or intraluminal stone enlargement within areas of intestinal stasis.

This case highlights the importance of considering non-fistulous gallstone ileus, even when classical radiologic findings are incomplete. Computed tomography remains the diagnostic modality of choice, allowing accurate identification of ectopic gallstones and obstruction sites. Surgical management with enterolithotomy alone is generally safe and effective, particularly in elderly patients, as additional biliary surgery is usually unnecessary in the absence of a fistula.

Recognition of this rare variant is essential to ensure timely diagnosis and appropriate surgical management in patients with small bowel obstruction and a history of gallstone disease.

## Introduction

Gallstone ileus is an uncommon but well-recognized cause of mechanical intestinal obstruction. It occurs in 0.3% to 0.5% of all patients with gallstones, in less than 0.1% of all cases of mechanical obstruction, and in 1% to 4% of non-strangulating mechanical small bowel obstructions. Mortality remains high, ranging from 12% to 27%, partially because of nonspecific symptoms, unremarkable biochemical investigations, a high misdiagnosis rate, and delayed diagnosis, accounting for up to 25% of cases among elderly patients [1,2]. The condition typically develops as a late complication of long-standing cholelithiasis, in which chronic inflammation of the gallbladder promotes adhesion to adjacent bowel loops and eventual formation of a bilioenteric fistula, most frequently cholecystoduodenal, allowing the passage of large gallstones into the gastrointestinal tract [3]. Once within the intestinal lumen, stones larger than 2-2.5 cm may become impacted, most commonly in the terminal ileum due to its relatively narrow diameter and decreased peristaltic activity [4].

The classical diagnostic paradigm relies on Rigler’s triad - intestinal obstruction, pneumobilia, and an ectopic gallstone - which reflects the presence of a biliary-enteric communication [5]. However, gallstone ileus occurring without formation of a bilioenteric fistula represents an exceptionally rare entity that challenges traditional pathophysiological concepts. Only isolated reports and small case series have described this presentation, suggesting alternative mechanisms of stone migration or intraluminal formation [6-8].

Because the absence of pneumobilia or fistula may obscure diagnosis, recognition of this atypical variant is essential to avoid delays in management. We present a case of gallstone ileus without evidence of bilioenteric fistula and discuss current theories explaining this rare phenomenon.

## Case presentation

A 74-year-old female with a known history of systemic arterial hypertension, chronic venous insufficiency, and mixed anxiety and depression disorder presented to the Emergency Department with colicky pain of 12 hours’ duration in the umbilical region following the ingestion of a banana smoothie. The pain progressively intensified over time, initially intermittent and later becoming continuous. It began as localized in that area and subsequently radiated to both iliac fossae and the suprapubic region. The condition was accompanied by 10 episodes of vomiting with gastric content. The patient denied nausea, fever, or any other associated symptoms when questioned. No past medical history of gallstone disease or previous abdominal surgery was mentioned by the patient or any family member.

On admission, the patient was hemodynamically stable but mildly tachycardic. Physical examination revealed a globose, distended abdomen due to adipose panniculus, with present but decreased peristalsis and tympany on percussion. On palpation, severe pain was noted in the umbilical and suprapubic regions, with positive rebound tenderness in both areas and involuntary muscular rigidity, which, together with the patient’s clinical history, is consistent with an acute abdomen. Laboratory evaluation demonstrated leukocytosis (12,400/mm³), predominantly due to neutrophilia (75.6%), and a glucose level of 135 mg/dL, with preserved renal and liver function tests. Arterial blood gas analysis demonstrated respiratory alkalosis (Table 1).

**Table 1:** Patient Laboratory Results pH: Potential of hydrogen; PaCO_2_: Partial pressure of arterial carbon dioxide; PaO_2_: Partial pressure of arterial oxygen; HCO_3_^-^: Serum bicarbonate; SatO_2_: Arterial oxygen saturation

Parameter	Result	Reference range
Complete blood count		
Leukocytes	12,400 /mm^3^	4.50-10.00
Neutrophils	75.6%	41.4-73
Blood chemistry panel		
Glucose	137 mg/dL	70-110
Arterial blood gas		
pH	7.52	7.35-7.45
PaCO_2_	28 mmHg	35-45
PaO_2_	95 mmHg	80-100
HCO_3_^-^	22 mEq/L	22-26
SatO_2_	98%	>95%

**Figure 1:**
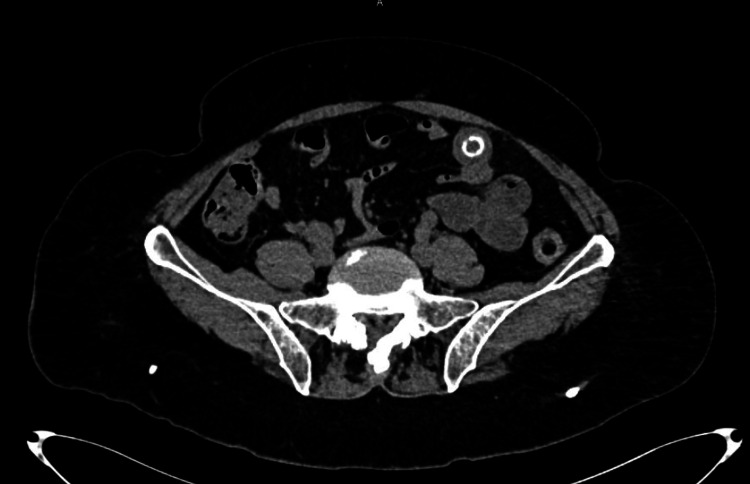
Axial View of Abdominal CT Scan A hyperdense image consistent with a gallstone measuring 13.2 mm × 22.1 mm was observed, with dilation of the small bowel loops. CT: computed tomography

**Figure 2:**
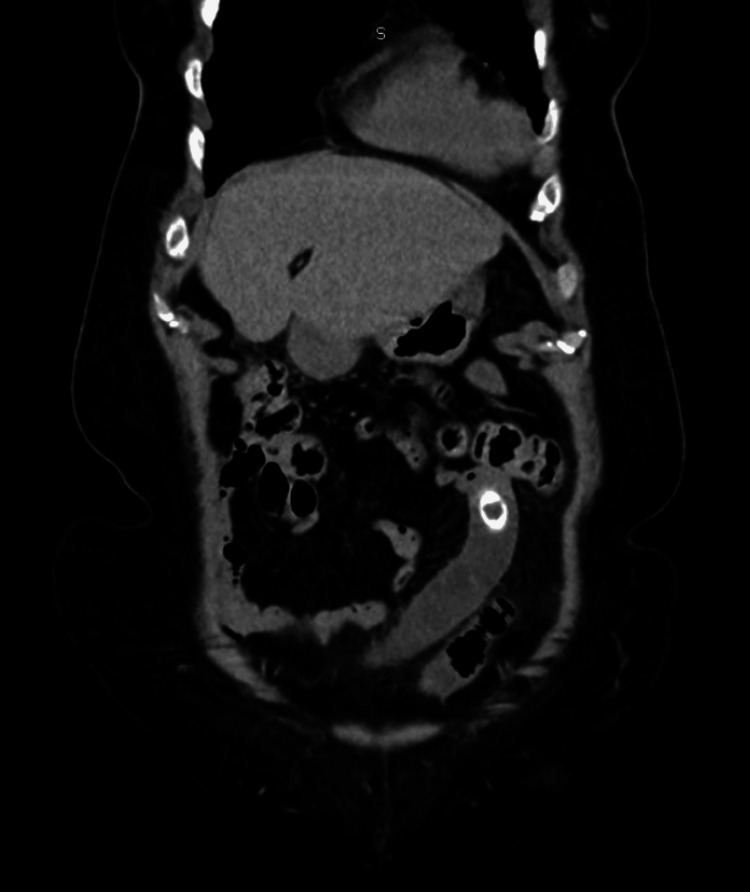
Coronal View of Abdominal CT Scan A hyperdense image consistent with a gallstone measuring 13.2 mm × 22.1 mm was observed, with dilation of the small bowel loops. CT: computed tomography

A non-contrast abdominal computed tomography (CT) scan was performed, revealing a hyperdense image consistent with a gallstone measuring 13.2 mm × 22.1 mm, with dilation of the small bowel loops distal to it (Figures 1-2).

**Figure 3:**
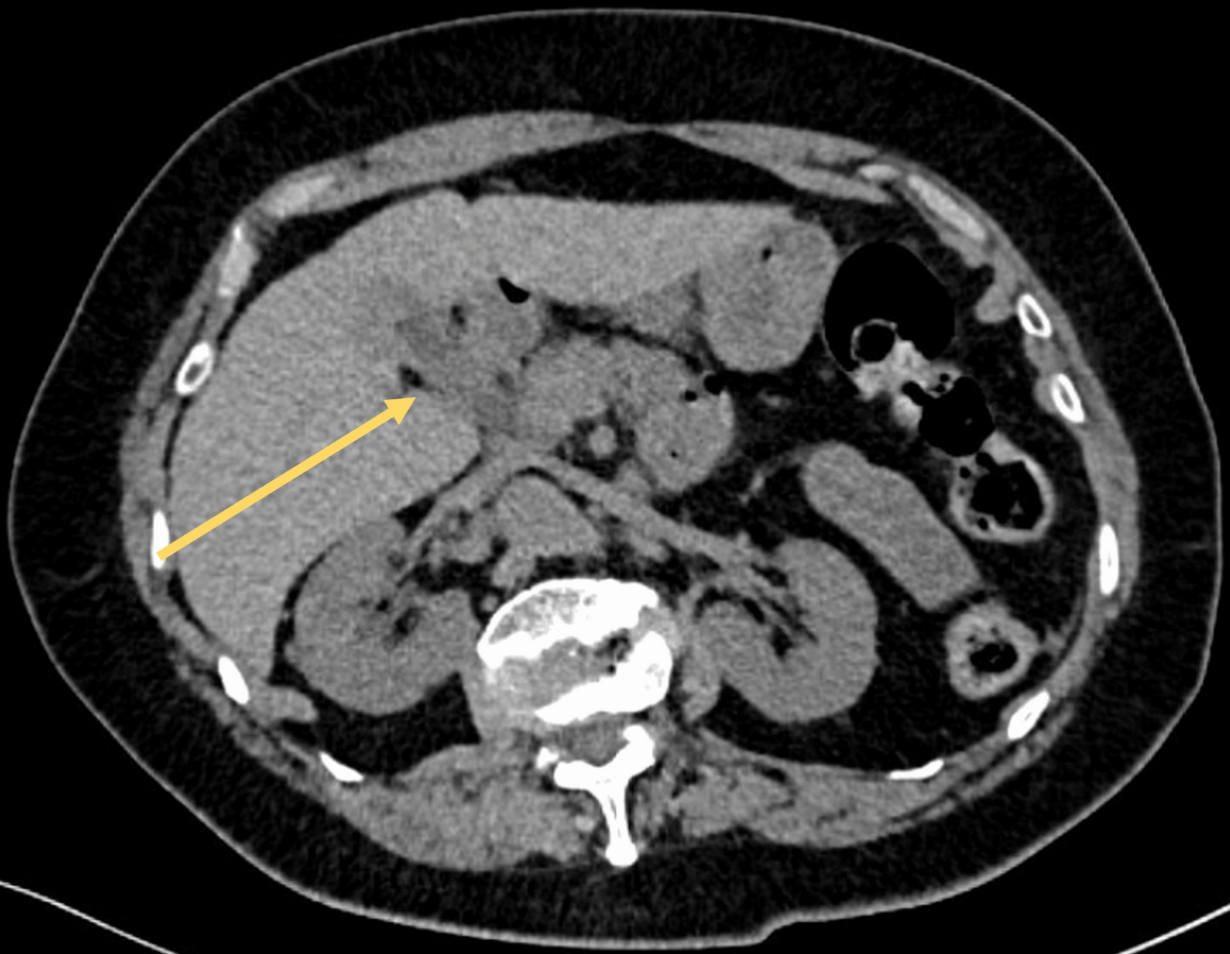
Axial View of Abdominal CT Scan The gallbladder and the common bile duct are observed to be free of air (yellow arrow), indicating the absence of air due to pneumobilia or a bilioenteric fistula. Additionally, the absence of gallbladder stones is observed. CT: computed tomography

Additionally, the gallbladder is visualized without the presence of gallstones, as well as without pneumobilia or a biliary-enteric fistula (Figure 3). These findings suggested mechanical small bowel obstruction caused by an ectopic gallstone, despite incomplete radiologic criteria for classical gallstone ileus.

After adequate fluid resuscitation and nasogastric decompression, an exploratory laparotomy was performed. Following asepsis and antisepsis of the abdominal region and placement of sterile drapes, a supra- and infraumbilical midline incision was performed in layers until entering the abdominal cavity. A systematic exploration was carried out, identifying dilated small-bowel loops. The site of obstruction was located in the ileum, 150 cm from the ileocecal valve and 140 cm from the ligament of Treitz, where an impacted gallstone measuring 2.5 cm in diameter was palpated. A nasogastric tube was placed, and retrograde intestinal decompression was performed.

It is important to note that no bilioenteric fistula was identified intraoperatively. A thorough and systematic exploration of the hepatobiliary region was performed, including careful inspection and palpation of the gallbladder (which contained gallstones), the duodenum, and adjacent bowel segments. The gallbladder wall appeared intact, without evidence of inflammation, adhesions, fibrosis, or abnormal communication with the gastrointestinal tract. There were no dense adhesions between the gallbladder and the duodenum or other bowel loops, which are typically observed in cases of chronic inflammation leading to fistula formation. Additionally, no visible tract, defect, or area of induration suggestive of a prior or active fistulous connection was identified. The absence of pneumobilia on preoperative imaging further supports this finding. Based on these intraoperative findings, the presence of an active bilioenteric fistula was reasonably excluded. The origin of the ectopic gallstone may be related to a transient or previously sealed microfistula not detectable at the time of surgery or to an alternative mechanism of migration. Nonetheless, given the presence of gallstones, a cholecystolithotomy was performed through an incision in the gallbladder fundus, and the stones were extracted. The gallbladder cavity was irrigated and subsequently closed using 2-0 Vicryl suture.

**Figure 4:**
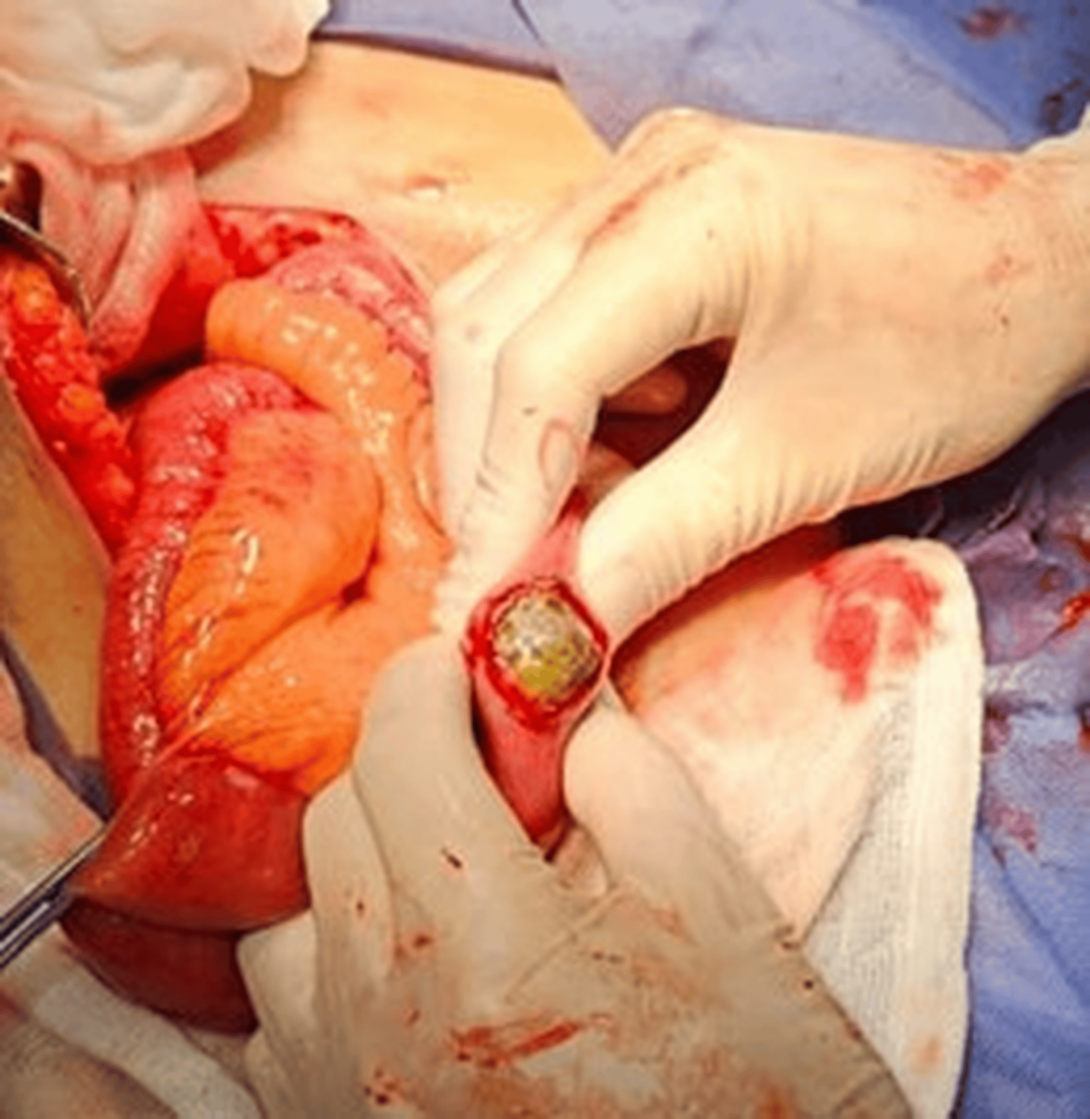
Removal of Gallstone in Small Bowel A longitudinal enterotomy was performed along the antimesenteric border, and the gallstone was removed without complications.

The different portions of the intestine were examined, identifying only a single stone. The affected intestinal segment was isolated using moist laparotomy pads, and a longitudinal enterotomy was performed along the antimesenteric border; the gallstone was removed without complications (Figure 4). Subsequently, the enterotomy was closed transversely in two layers (mucosal and seromuscular) using the Heineke-Mikulicz technique with 3-0 Prolene sutures, employing Connell-Mayo stitches and Lembert inverting sutures. Adequate intestinal lumen patency and absence of leakage were confirmed. Hemostasis was verified, and a half-inch Penrose drain was placed in the surgical bed. Sponge and instrument counts were performed. Layered closure was completed: the aponeurosis was closed in a single layer with PDS 1-0, subcutaneous tissue with Vicryl 1-0, and skin with nylon using subdermal stitches. The wound was covered with sterile gauze and dressings. The patient was transferred to recovery with stable vital signs.

Known imaging limitations can explain the absence of gallstones on preoperative CT. Although CT is considered the gold standard for the diagnosis of gallstone ileus, its sensitivity for detecting gallstones varies depending on their composition. Cholesterol-rich stones or those with low calcium content may appear isoattenuating relative to surrounding bile, rendering them difficult to identify, particularly in non-contrast studies. Additionally, small stones, overlapping bowel contents, fluid-filled gallbladder, and partial volume effects may further obscure their visualization. In this case, these factors likely contributed to the failure to detect gallbladder calculi preoperatively despite their intraoperative confirmation.

The postoperative course was uneventful, with recovery of bowel function on postoperative day 4 and progressive advancement of oral intake. The patient was discharged on postoperative day 5 in stable condition and remained asymptomatic at follow-up evaluation.

## Discussion

Gallstone ileus represents a diagnostic and therapeutic challenge primarily affecting elderly patients with significant comorbidities, contributing to reported mortality rates ranging between 12% and 27% [2,4]. Classical pathogenesis involves chronic inflammation leading to pressure necrosis and fistula formation between the gallbladder and gastrointestinal tract, allowing migration of large gallstones incapable of passing through the cystic duct or common bile duct under normal conditions [3]. The present case is notable because intestinal obstruction occurred without evidence of a bilioenteric fistula, a phenomenon rarely described in surgical literature [8]. Several mechanisms have been proposed to explain this atypical presentation.

One hypothesis involves transpapillary migration, whereby gallstones pass through the common bile duct and ampulla of Vater into the duodenum. Although uncommon, dilation of the papilla or transient obstruction may permit passage of relatively large calculi, particularly in elderly patients with biliary tract remodeling [6]. Similar cases have been reported following endoscopic sphincterotomy, in which enlargement of the papillary orifice facilitates stone migration into the bowel lumen [7]. Another proposed mechanism is primary enterolith formation, in which small biliary fragments act as a nidus for progressive accretion within areas of intestinal stasis, such as diverticula or segments affected by motility disorders [9]. Altered bile salt metabolism and bacterial overgrowth may contribute to stone enlargement after entering the intestinal tract. Additionally, some authors suggest that previously existing microfistulas may close spontaneously before surgical exploration, leaving no detectable communication at the time of operation [9]. This theory could explain cases lacking pneumobilia, despite presumed prior biliary-enteric passage. From a diagnostic perspective, the absence of pneumobilia significantly reduces clinical suspicion because Rigler’s triad (pneumobilia, ectopic gallstone, and small bowel obstruction) is incomplete.

CT remains the most sensitive imaging modality, with diagnostic accuracy exceeding 90%, even when classical features are absent [5]. Identification of an intraluminal calcified mass associated with a transition point should prompt consideration of gallstone ileus regardless of fistula visualization. Even so, the differential diagnoses for gallstone ileus include acute pancreatitis, bile duct stones, cholecystitis, bile duct malignancy, or peptic ulcer disease; in this particular case, we may also include bezoar obstruction or a calcified tumor [6].

Management strategies remain similar to conventional gallstone ileus. In the present case, enterotomy with stone extraction was performed as the primary procedure, as the patient belonged to a high surgical risk group due to her age (74 years) and the presence of significant comorbidities, including systemic arterial hypertension and chronic venous insufficiency. In this context, current evidence supports enterolithotomy alone as the preferred approach in elderly or comorbid patients, as it allows prompt resolution of intestinal obstruction with shorter operative time, reduced physiological stress, and lower complication rates.

Several studies have shown that more extensive procedures, such as one-stage surgery (enterolithotomy combined with cholecystectomy and fistula closure), are associated with higher morbidity and a potential increase in mortality and, therefore, should be reserved for younger, stable patients with low operative risk. In contrast, in elderly patients, the primary goal is the rapid relief of intestinal obstruction, making enterolithotomy alone the most widely accepted strategy. Additionally, in this case, cholecystolithotomy was performed as an adjunct procedure without cholecystectomy during the same surgical stage. This decision was based on the absence of a prior history of cholecystitis or biliary symptoms, as well as imaging findings showing a gallbladder without residual stones, which reduces the likelihood of recurrence or future biliary complications. Recent literature suggests that the absence of residual gallstones may justify avoiding additional biliary procedures during the initial surgery, particularly in high-risk patients. Furthermore, it has been described that even in the presence of a biliary-enteric fistula, spontaneous closure may occur in a significant proportion of cases, making its repair unnecessary during the initial operation, especially in frail patients. On the other hand, in cases without a fistula, definitive biliary surgery is generally unnecessary, further supporting a limited surgical approach [1,4,6,10,11]. The following table compares one-stage surgery versus two-stage surgery (Table 2). 

**Table 2:** Comparison Between One-Stage Surgery Versus Two-Stage Surgery in Elderly Patients

Stage of treatment	Objective	One-stage surgery (enterolithotomy + cholecystectomy + fistula closure)	Two-stage surgery (enterolithotomy → delayed surgery)	Considerations in elderly patients
Resuscitation and initial management	Hemodynamic stabilization, fluid and electrolyte correction	Same in both approaches	Same in both approaches	Essential due to high comorbidity and frequent dehydration [10]
Resolution of intestinal obstruction (acute phase)	Relieve intestinal obstruction	Enterolithotomy + biliary surgery in the same procedure	Enterolithotomy alone (shorter procedure)	In elderly patients, shorter procedures are preferred due to lower surgical stress [1]
Management of biliary pathology (gallbladder and fistula)	Prevent recurrence and biliary complications	Managed in the same surgical stage	Deferred (4 weeks-6 months)	In elderly patients, often omitted or delayed due to surgical risk [1]
Risk of recurrence	Prevent recurrent gallstone ileus	Low (~1%)	Higher (≈2-8%)	Higher recurrence may be acceptable if it reduces initial mortality [10]
Surgical morbidity	Reduce postoperative complications	Higher (longer and more complex surgery)	Lower	Especially important in frail patients or those with comorbidities [1]
Mortality	Survival	Similar or slightly higher in some series	Lower in several studies	Enterolithotomy alone shows lower mortality in cohorts [11]
Operative time and invasiveness	Physiological impact	Longer and more invasive	Shorter and less invasive	Key factor in elderly patients (limited physiological reserve) [1]
Ideal indications	Patient selection	Stable patients, low risk, good physiological reserve	Unstable patients, high risk, or frail elderly	Choice depends more on clinical status than on the disease itself [1]
Long-term complications	Biliary sequelae	Lower risk of gallbladder cancer or recurrent cholecystitis	Persistent fistula (theoretical cancer risk up to 15%)	In elderly patients, long-term impact is usually less relevant than immediate survival [10]

Recurrence after isolated enterolithotomy is uncommon, reported in fewer than 10% of patients, and spontaneous resolution of biliary pathology frequently occurs [2].

Overall, the management strategy adopted in this case is consistent with current evidence, which emphasizes an individualized approach, prioritizing patient stability and reserving more complex procedures for selected cases.

## Conclusions

Gallstone ileus without a bilioenteric fistula is an extremely rare variant of an already uncommon disease and represents a diagnostic challenge due to the absence of classical imaging findings, such as pneumobilia. Alternative mechanisms, including transpapillary migration or intraluminal stone growth, should be considered when evaluating small bowel obstruction in patients with known gallstone disease. Early recognition through CT and prompt surgical treatment with enterolithotomy remains essential for favorable outcomes. Awareness of this atypical presentation may prevent diagnostic delay and improve patient prognosis.
